# Serum α-Glucosidase Activity as a New Parameter of Negative Energy Balance in Dairy Cows

**DOI:** 10.3390/vetsci13020122

**Published:** 2026-01-27

**Authors:** Babett Bartling, Thomas Tröbner, Lena Grone, Marion Schmicke

**Affiliations:** 1 Institute of Agricultural and Nutritional Sciences, Sustainable Livestock Husbandry and Animal Health Management, Martin Luther University Halle-Wittenberg, 06120 Halle (Saale), Germany; 2 Office for Agriculture, Land Consolidation, and Forestry, Agriculture Department Altmark, 29410 Salzwedel, Germany; 3 University of Veterinary Medicine Hannover, Clinic for Cattle, Veterinary Endocrinology and Laboratory Diagnostics, 30559 Hannover, Germany

**Keywords:** assay, blood, enzyme activity, metabolism, parity

## Abstract

Enzymes of the α-glucosidase group cleave α-D-glucose from the non-reducing end of short oligosaccharides. They contribute to carbohydrate digestion as maltase-glucoamylase in the intestinal brush border and as neutral α-glucosidase in other tissues. Neutral α-glucosidase is also active in blood, but little is known about its relevance as an indicator of the body’s metabolic state. Therefore, we proved whether the α-glucosidase activity level in blood does reflect the state of negative energy balance (NEB). As NEB commonly occurs in dairy cows around calving, our study included blood (serum, plasma) samples of 73 Holstein Friesian cows collected ±14 d to parturition. After the establishment and characterization of a fast and low-cost activity assay, these blood samples were analyzed for α-glucosidase compared to known NEB biomarkers. This analysis revealed the lowest α-glucosidase activity 5 d *post partum* (−25% compared to 14 d *ante partum*) by using two different α-glucosidase substrates. The reduced activity recovered 14 d *post partum*; however, the degree of recovery depended inversely on the number of parities. In this regard, α-glucosidase activity changed peripartum in line with known biomarkers (e.g., NEFA, IGF-1, glucose). In conclusion, the α-glucosidase activity is a new and easily detectable blood parameter of NEB in dairy cows.

## Simple Summary

Dairy cows suffer from a calorie deficit, i.e., negative energy balance (NEB), around calving because their bodies expend more calories than they consume. Various biomarkers are routinely recorded on farms or in scientific studies to assess the extent of NEB. Our present study identified and described a new parameter that also effectively reflects the body’s metabolic state in dairy cows. This parameter is called neutral α-glucosidase. Neutral α-glucosidase belongs to the enzyme group of α-glucosidases that cleave glucose, the most abundant sugar, from short oligosaccharides. Its activity is detectable by a fast and low-cost enzymatic assay. Our study established and characterized this assay for bovine blood (serum, plasma) samples.

## 1. Introduction

The enzyme group of α-D-glucoside glucohydrolases, α-glucosidases in short, is essential for carbohydrate metabolism. The α-glucosidases hydrolyze the terminal, non-reducing α-linked α-D-glucose residue of di- and short oligosaccharides with subsequent release of α-D-glucose, the most abundant sugar. In vertebrates, α-glucosidases can be simply grouped into intestinal, lysosomal, and endoplasmic α-glucosidases. The intestinal α-glucosidases are maltase-glucoamylase and sucrase-isomaltase [1]. Both are membrane-bound enzymes in the brush border of enterocytes, where they synergistically digest carbohydrates from food [1]. The lysosomal α-glucosidase contributes to glycogenolysis in the acidic lumen of lysosomes, and it is therefore also called acid α-glucosidase [2]. Its deficiency leads to Morbus pompe, a rare glycogen storage disease [3]. In addition to lysosomes, the endoplasmic reticulum (ER) of cells contains α-glucosidase activity. Endoplasmic glucosidase activity is based on the expression of the α-glucosidases I and II and contributes to the biosynthesis of N-linked glycoproteins in the ER lumen. Although both glucosidases require neutral pH conditions and can be detected in nearly all cells and tissues, only α-glucosidase II is called neutral or tissue α-glucosidase [4,5], whereas α-glucosidase I corresponds to the mannosyl-oligosaccharide glucosidase [6,7].

Neutral α-glucosidase activity is commonly detected in enzyme assays with α-D-glucopyranoside coupled to either 4-nitrophenol [8], 6-bromo-2-naphthol [9], 2-naphthol [10], 4-methylumbelliferon [11], or resorufin [12]. Most commercial assays use either chromogenic 4-nitrophenyl α-D-glucopyranoside (4-NPG) or fluorogenic 4-methylumbelliferyl α-D-glucopyranoside (4-MUG) as α-glucosidase substrates. These short synthetic glucosides can only be hydrolyzed by α-glucosidase II because α-glucosidase I requires oligosaccharides of at least three monosaccharides [6,13,14]. Therefore, the neutral α-glucosidase activity measured in cells and tissues largely corresponds to the activity of α-glucosidase II [4,15]. Performed at acidic pH conditions, enzymatic assays using α-D-glucopyranoside substrates also measure the lysosomal α-glucosidase activity [11,12].

In addition to cells and tissues, neutral α-glucosidase activity can be detected extracellularly [16,17,18,19,20,21]. As neutral α-glucosidases contribute to the N-glycosylation of proteins, an important post-translational modification of most extracellular proteins, they are probably released into the extracellular space together with these proteins by exocytosis [22]. In this regard, extracellular α-glucosidase activity has been found in urine [16,19], ejaculate [8], and blood serum [17,20,21,23]. Quantifications in ejaculate samples are based on a standardized WHO protocol because it is a common part of the investigation into the cause of male infertility [24]. As α-D-glucose selectively inhibits the neutral α-glucosidase activity (IC_50_ at about 2 mmol/L) [8], the glucose content in biological samples should always be determined as well for better data interpretation.

The hydrolytic activity of the neutral α-glucosidase in blood fluid could probably also be used for diagnostic purposes in the veterinary field. In this regard, Kotonska-Feiga et al. (2015) identified seasonal changes in the serum neutral α-glucosidase levels of breeding carps with the highest values in summer and the lowest after the winter period [20]. Moreover, we recently identified breeding-dependent differences in the serum neutral α-glucosidase of meat cattle with higher activity values for cattle kept intensively compared to cattle kept extensively [21]. Although these observations suggest a correlation between the general metabolic activity of the body and α-glucosidase activity in blood fluid, it is unclear how quickly the serum α-glucosidase activity changes in response to a change in the body’s metabolic state.

To gain more clarity about the dependence of the serum α-glucosidase activity on metabolic activity, blood samples collected during the perinatal period of dairy cows might be ideal study samples. Nearly all dairy cows experience a period of negative energy balance (NEB) after parturition because the energy demand for milk production during early lactation is not compensated for by the energy intake. Therefore, the cow mobilizes the body’s own energy depots, especially body fat, to sustain her milk production [25,26]. This compensatory mechanism leads to a lower body condition score (BSC) and changes in defined metabolic parameters [25,26,27]. Typical changes are an increase in free fatty acids (non-esterified fatty acids (NEFA)) and ketone bodies (β-hydroxybutyrate (BHB)) in blood, whereas carbohydrate parameters (glucose, fructosamine), hormones of the somatotropic axis (insulin-like growth factor (IGF)-1), pancreatic insulin, and, if not sufficiently compensated, calcium are decreased [25,26,27]. Moreover, intensive research in this field has led to the identification of further factors and therefore NEB-related biomarkers (e.g., liver-derived fibroblast growth factor 21, adipocyte tissue-derived leptin, and infrared-spectroscopy-determined milk composition) in recent years [28,29,30,31].

We suspect that neutral α-glucosidase activity could be another new and interesting blood parameter for describing the general metabolic activity in livestock. For this reason, our study aimed to investigate α-glucosidase activity in the perinatal period in dairy cows and compare it with known metabolic parameters.

## 2. Materials and Methods

### 2.1. Study Population and Sample Collection

This study analyzed blood samples from 73 dairy cows (no heifers) of the breed Holstein Friesian. All cows were kept on a farm in Germany in the federal state of Saxony-Anhalt. They received standard feed (wilted silage, maize silage, chopped straw, rapeseed, rye, or corn meal) supplemented with mineral feed (HL Hamburger Leistungsfutter GmbH, Hamburg, Germany) and fresh water. In order to prevent the risk of clinical ketosis in the perinatal period, cows additionally received propylene glycol that had been poured over the food. In case of a body condition score (BCS) ≥ 4.25 during the cow’s dry period, the individual cow was co-treated with a Kexxtone^®^ bolus (Elanco, Indianapolis, IN, USA). The prophylactic treatment of parturient paresis was carried out with Calcitat^®^N25 (aniMedica GmbH, Senden, Germany) and a calcium-containing bolus (various providers).

Blood samples were collected from each cow at four different time points: 14 d *ante partum* according to the estimated date of calving (13 ± 4 d according to the actual time of calving), on the day of calving or the following day, 5 d *post partum*, and 14 d *post partum*. Blood was always taken from the caudal vein into serum and K_3_-EDTA blood collection tubes (S-Monovette; Sarstedt, Nümbrecht, Germany) in the morning (08:00 to 11:00 h), gently mixed, and then centrifuged at 2000× *g* (10 min) to remove blood clots and cells. The resulting serum and plasma samples were aliquoted into small reaction tubes and stored at −20 °C until analysis. Moreover, we recorded various basic parameters of the cow, including BCS, number of parities, and perinatal complications.

### 2.2. Standard Blood Parameters

Blood parameters more commonly assessed in dairy cows were measured by the following test systems. BHB-check β-keton strips (Pharmadoc GmbH, Lüdersdorf, Germany) quantified the BHB concentration in whole blood. A calcium test kit quantified protein-bound calcium in serum samples by the o-cresolphtalein complex formation method (Diaglobal GmbH, Berlin, Germany). A two-step enzymatic test kit quantified NEFA in serum samples compared to an oleic acid concentration standard (Diaglobal GmbH). Colorimetric ELISA tests for IGF-1 (E20; Mediagnost GmbH, Reutlingen, Germany) and insulin (#10-1201-01; Mercodia, Uppsala, Sweden) were used to quantify either blood parameter in serum samples.

In addition, we analyzed carbohydrate-related blood parameters. Serum glucose was quantified by the glucose oxidase method using a commercial test kit (GAGO20; Sigma, St. Louis, MO, USA). Serum fructosamine was assessed by an improved nitro-blue tetrazolium-based method, as described previously [21].

### 2.3. α-Glucosidase Assay

The α-glucosidase activity was quantified in blood samples using the enzyme substrates 4-nitrophenyl α-D-glucopyranoside (4-NPG; Thermo Fisher Scientific Inc., Waltham, MA, USA) and 4-methylumbelliferyl α-D-glucopyranoside (4-MUG; Carl Roth GmbH, Karlsruhe, Germany). 4-NPG is cleaved into α-D-glucose and 4-nitrophenol (4-NP), whereas 4-MUG is cleaved into α-D-glucose and 4-methylumbelliferone (4-MU). As blood samples, we used serum and plasma with or without repeated freeze–thaw cycles and with or without experimental hemolysis. Repeated freeze–thaw cycles were generated by freezing the serum or plasma samples at −20 °C with subsequent thawing at 37 °C. Experimental hemolysis was reached by adding a defined volume of lysed whole blood to the serum or plasma sample. These volumes were 2 and 4%, which corresponds to an approximate Hb concentration of 0.25 and 0.5 g/dL, respectively.

The enzymatic assay was performed in triplicate as follows. A 20 µL blood sample was added to a well of a 96-well microplate and mixed with 180 µL reaction buffer containing the particular substrate (1 mmol/L after preliminary analysis) in McIlvaine (citrate-phosphate) buffer (adjusted to pH 6.8 or 7.4). While the rate of 4-NP formation was continuously monitored at 405 nm for 30 min (37 °C) in a microplate reader, the continuous 4-MU formation was monitored at 347 nm. In the case of 4-MU, the transmission of the microplates for UV-A light had been ensured. Negative control reactions with an α-glucosidase inhibitor were performed by adding acarbose (Thermo Fisher Scientific Inc.) to a final concentration of 40 µmol/L. All measurements were performed using the computer-controlled FLUOstar Omega reader equipped with MARS data analysis software (version 3.32 R5; BMG Labtech, Ortenberg, Germany). The amount of α-glucosidase necessary to form 1 µmol of 4-NP from 4-NPG (or 4-MU from 4-MUG) per minute is defined as one unit of enzymatic activity (U). The enzymatic activity was then calculated using the respective molar extinction coefficient (ε) and a path length of 0.58 cm in a 200 µL-filled well. All ε values were separately determined for 4-NP and 4-MU (each Carl Roth GmbH) in McIlvaine buffer at pH 6.8 and 7.4 (n = 4 test series). According to our measurement conditions, the McIlvaine buffer was supplemented with 10% bovine blood sample. The presence of serum or plasma in the McIlvaine buffer slightly increased the ε values for 4-MU, but it had no effect on the values for 4-NP. We identified the following ε values (cm^−1^ mmol/L^−1^): 6.76 for 4-NP_pH 6.8_, 13.26 for 4-NP_pH 7.4_, 7.17 for 4-MU_pH 6.8_, and 9.98 for 4-MU_pH 7.4_.

### 2.4. Statistics

Data presentations and statistical analyses were performed using the OriginPro 2019 software (OriginLab Corporation, Northampton, MA, USA). Data are given as stated in table footnotes and figure legends. In a few cases, it was not always possible to determine all serum parameters for the 73 cows of our population study. The normality of data sets was tested by the Shapiro–Wilk test. Unless otherwise indicated, differences between the evaluation groups were tested for significance using the paired Student’s *t*-test (parametric), Wilcoxon signed-rank test (non-parametric), Mann–Whitney U test (non-parametric), standard or repeated measures (rm) ANOVA statistics with the Bonferroni post hoc procedure (parametric) or ANOVA on Ranks statistics (non-parametric), or the chi^2^ test. *p*-values below 0.05 indicate significant differences. The statistical tests used in each case are always mentioned in the table footnotes and figure legends.

## 3. Results

Our study consists of two parts. The first part describes the establishment of the α-glucosidase assay for bovine blood samples. Here, we used blood samples of a small number of cows only. The second part presents the results of the perinatal α-glucosidase activity in blood samples collected from a larger population of dairy cows. The term α-glucosidase used in this section always refers to the neutral variant of the α-glucosidase.

### 3.1. Assay Establishment

The enzymatic activity of the α-glucosidase was comparatively measured using the synthetic glucoside substrates 4-NPG and 4-MUG under different conditions. In contrast to 4-NPG, which is used in chromogenic assays, 4-MUG is commonly used in fluorometric assays. As the hydrolysis of 4-MUG can also be detected chromogenically [32], we used both substrates as chromogens and a final concentration of 1 mmol/L of each substrate. This concentration proved to be appropriate for blood samples in preliminary tests. Adding α-glucosidase-inhibitory acarbose always prevented the hydrolysis of 4-NPG or 4-MUG completely, an observation we had already made before [21].

**Figure 1:**
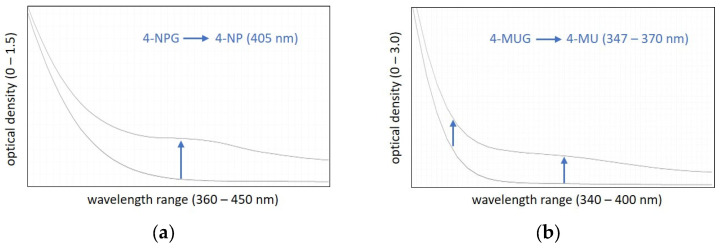
Absorbance spectrum of the α-glucosidase substrates 4-NPG (**a**) and 4-MUG (**b**) in a bovine serum sample before and after enzymatic cleavage into 4-NP and 4-MU, respectively.

The α-glucosidase-mediated hydrolysis of 4-NPG leads to the continuous formation of 4-NP, which can be detected in the visible range at 405 nm (Figure 1a). The 4-MUG hydrolysis leads to the formation of 4-MU, which is, in contrast to 4-NP, only detectable in the UV-A range. Van Tilbeurgh et al. (1988) originally suggested the use of 347 nm for 4-MU detection [32]. Our preliminary tests confirmed this suggestion. The 4-MU spectrum (maximum at 324 nm) does not overlap at 347 nm any more with that of the 4-MUG substrate (spectrum not shown). We also showed that a wider UV-A range (−370 nm) is possible to detect the 4-MU signal (Figure 1b). However, the change in absorption per unit of time (ΔOD_4-MU_) proved to be slightly better at 347 nm than at higher wavelengths.

**Figure 2:**
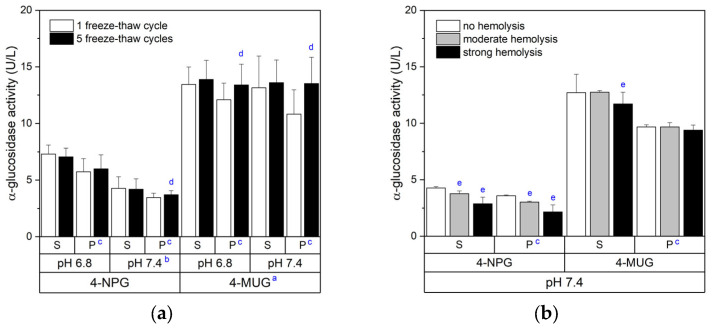
Bovine α-glucosidase activity in blood serum (S) or plasma (P), depending on the enzyme substrate, pH value of the assay buffer, and number of the sample freeze–thaw cycles (**a**), as well as depending on the hemolysis in blood samples (**b**). Serum and plasma samples were always selected from the same cow and sampling time (n = 10). Hemolytic samples contained whole blood to a final concentration of 2% (moderate) or 4% (strong). Data are presented as mean ± SD with *p* ≤ 0.05 for differences between the ^a^ substrates, ^b^ pH values, ^c^ blood sample types, ^d^ freeze–thaw cycles, and ^e^ degree of hemolysis under otherwise identical conditions (paired *t*-test vs. respective internal control).

The enzymatic activity can be calculated from the ΔOD values using a standard series of recombinant α-glucosidase (Myozyme^®^ or Lumizyme^®^ as human α-glucosidase) or using standard series or ε values of the respective chromophores. We decided to use the ε values of the chromophores (4-NP or 4-MUG) at given measuring conditions because it does not require an additional standard series on the microplate. As pH values of the assay buffer, we choose 6.8 and 7.4. pH values around 6.8 are more commonly used in α-glucosidase assays [8,9,18,20,21,24], whereas pH 7.4 refers to the physiological pH value in blood plasma. In this regard, we additionally compared the α-glucosidase activity between plasma and serum samples and between samples that had been repeatedly frozen and thawed. These initial experiments showed an influence of the particular α-glucosidase substrate, the pH value of the assay buffer, and the type of blood samples on the α-glucosidase activity values, whereas repeated freeze–thaw cycles were of less importance (Figure 2a). The α-glucosidase activity values assessed with the 4-MUG chromophore were generally higher than the values assessed with 4-NP (Figure 2a). This chromophore-dependent difference was already visible directly at the photometer (ΔOD per time). Independent of the chromophore, serum samples always showed slightly higher α-glucosidase activity values than plasma samples (Figure 2a). A clear impact of the pH value had only been determined for the α-glucosidase activity assessed with 4-NP (Figure 2a). Repeated freeze–thaw cycles had no negative effect on the α-glucosidase activity (Figure 2a). In the case of plasma samples repeatedly frozen and thawed, the α-glucosidase activity was even slightly higher, especially when using 4-MUG as a substrate (Figure 2a). Technical differences within the 4-NPG or 4-MUG measurement series can be nearly excluded, as all samples prepared were analyzed together on one microplate.

During routine blood collection or at a later time point, erythrocytes can be damaged, which results in a so-called hemolytic sample. As hemolysis often impairs test assays, we also analyzed the effect of hemolysis on the α-glucosidase activity in serum and plasma samples. This analysis identified a negative impact of hemolysis when using 4-NPG as an α-glucosidase substrate, whereas it had no clear effect when using 4-MUG (Figure 1b).

### 3.2. Analysis of the Population

Our assay establishment described above showed the best α-glucosidase activity under the following conditions: serum as blood sample type, 4-MUG as enzyme substrate, and pH 7.4 (or 6.8) as buffering condition. Because 4-MUG is more cost-intensive than 4-NPG and, therefore, may be used less in practice, we measured the serum α-glucosidase activity in the perinatal period of dairy cows by using both substrates under otherwise identical conditions. The population of dairy cows studied was characterized by the typical changes in various metabolic parameters prior to and after parturition (Table 1). While the blood levels of BHB and NEFA increased *post partum*, the levels of IGF-1 and insulin decreased (Table 1). Moreover, the BCS moderately decreased by 0.3 ± 0.7 between both time points (*p* < 0.001), whereas the blood levels of calcium remained unchanged (2.32 ± 0.42 mmol/L).

**Figure 3:**
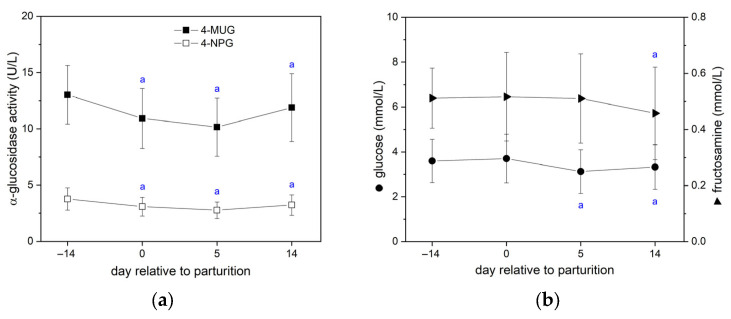
Bovine serum α-glucosidase activity in the perinatal period of dairy cows (**a**) compared with other carbohydrate-related serum parameters (**b**). α-glucosidase activity was detected comparatively with 4-NPG (405 nm) and 4-MUG (347 nm) at pH 7.4. Glucose and fructosamine were selected as carbohydrate-related parameters. Data are presented as mean ± SD (n = 73 cows) with ^a^ *p* < 0.05 vs. 14 d *ante partum* (rmANOVA with Bonferroni test). Further significances are present but are not indicated.

While blood parameters more commonly assessed in dairy cows were studied at two time points only (Table 1), the α-glucosidase activity and related carbohydrate parameters (glucose, fructosamine) were measured at all four time points of blood sampling (Figure 3). In this regard, we identified the highest serum α-glucosidase activity 14 d *ante partum* and lowest 5 d *post partum* with partial recovery at day 14 (Figure 3a). Although the enzymatic activity measured with 4-MUG was generally higher than with 4-NPG, both substrates identified the same perinatal changes (Figure 3a; Pearson correlation coefficient: 0.88 for all samples with *p* = 4 × 10^−90^). Only 5 of 73 cows (7%) showed no lowered α-glucosidase activity at day 5 *post partum* compared to the prenatal values. Like the serum α-glucosidase activity, the serum levels of glucose were lowest 5 d *post partum*, whereas the levels of fructosamine were lowest 14 d *post partum* (Figure 3b).

**Figure 4:**
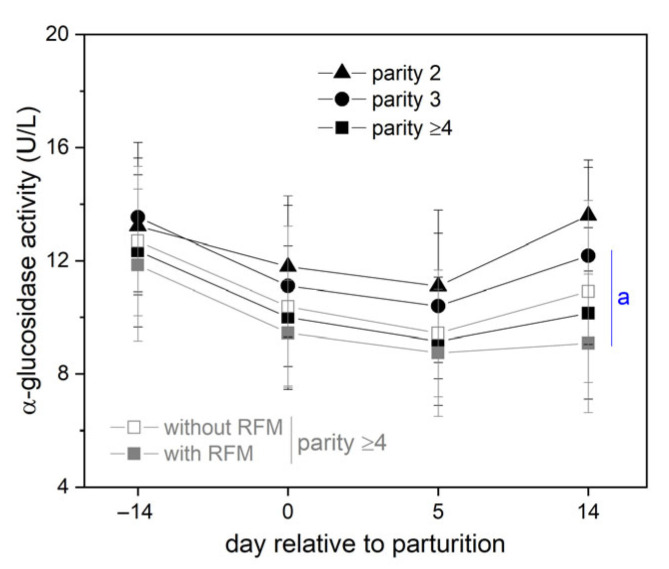
Bovine serum α-glucosidase activity in the perinatal period of dairy cows, depending on parities and depending on RFM complications in cows that were at least in their fourth parity. The enzyme activity given here was detected with 4-MUG (347 nm) at pH 7.4. Data are presented as mean ± SD (n each group according to Table 2) with ^a^ *p* < 0.05 for 14 d *post partum* vs. 14 d *ante partum* (rmANOVA with Bonferroni test each parity group). Further significances are present but not indicated.

During the postnatal period, various complications can occur, of which retained fetal membrane (RFM) was the most common in our study population, at 25% (17% mastitis, 16% parturient paresis/hypocalcemia (blood Ca ≤ 2 mmol/L), 12% low BCS (≤3), 4% endometritis, and 4% clinical ketosis (blood BHB ≥ 3 mol/L)). Cows with RFM complication also showed the lowest α-glucosidase activity *post partum*. As RFM mainly occurred in older, multiparous cows, we sub-grouped our study population according to the number of parities and according to the occurrence of RFM in cows that are at least in their fourth parity. This analysis showed comparable changes in the α-glucosidase activity during the perinatal period for all sub-groups (Figure 4). However, biparous cows were characterized by a full recovery of the serum α-glucosidase 14 d *post partum*, whereas this recovery steadily worsened with the number of further parities (Figure 4).

Multiparous dairy cows with at least four parities and RFM complication after their last calving showed the lowest mean serum α-glucosidase activity during the entire perinatal period, but there were high intra-individual differences (Figure 3). Therefore, Table 2 lists the more common and carbohydrate-related blood parameters only depending on the number of parities. In correspondence with the lowest recovery of the α-glucosidase activity in dairy cows with at least four parities, they were also characterized by the lowest postnatal blood values for IGF-1, glucose, and fructosamine and the highest value for NEFA (Table 2). Other parameters, including BCS and the frequency of hypocalcemia, did not depend on the number of parities and thus did not depend on the perinatal α-glucosidase activity.

## 4. Discussion

Our study identified the neutral α-glucosidase activity as a new blood parameter that effectively reflects the metabolic state of dairy cows. Using two different α-glucosidase substrates, we showed that the α-glucosidase activity is particularly low when the cow is in its NEB period around calving. Although the α-glucosidase activity recovered after birth, the degree of recovery depended inversely on the number of parities. The determination of the α-glucosidase activity in blood samples proved to be relatively robust and can be performed on both serum and plasma.

Various dye-coupled α-D-glucopyranoside substrates are available that can be used to quantify the α-glucosidase activity in biological samples. Glucopyranoside coupled with 4-NP is among the most common and longest-used substrates. Therefore, many commercial α-glucosidase activity assays and a laboratory WHO protocol [24] use 4-NP as a detection dye. However, 4-NP is not necessarily the best choice, as enzyme activity is often determined using the extinction coefficient, but in the case of 4-NP, this is highly pH-dependent [33]. This is one of the reasons why we used 4-MU, an alternative dye, to detect the α-glucosidase activity. 4-MU proved to be much less pH-dependent than 4-NP in our preliminary investigations. Unlike most 4-MU-based assays that use this dye as a fluorogen [11], we used 4-MU as a chromogen. This possibility was already proposed in the 1980s [32] but not really implemented in enzymatic assays afterwards. Our present study confirmed the usability of 4-MU as a chromogen to detect α-glucosidase activity. Moreover, we showed that the activity of the neutral α-glucosidase variant is higher in blood samples when analyzed with α-D-glucopyranoside coupled to 4-MU instead of 4-NP. A plausible explanation for this discrepancy may be a better fit of 4-MU-coupled substrate to the active site of the α-glucosidase. This might also explain why hemolysis or moderate changes in the neutral pH range had little or no effect on the α-glucosidase activity when determined with 4-MU-coupled α-D-glucopyranoside (4-MUG). Regardless of this, we were also able to show that the neutral α-glucosidase activity in serum and plasma samples is very robust and hardly dependent on how often frozen samples have been thawed again. In the case of plasma samples and 4-MUG as a substrate, we even observed that neutral α-glucosidase activity is higher in samples that have been frequently thawed and refrozen. Here, one or more inhibitory factors present in plasma but not in serum may be destroyed by multiple freeze–thaw cycles. In any case, plasma must contain glucosidase-inhibiting factors, as, regardless of the enzyme substrate (4-NPG or 4-MUG), α-glucosidase activity in plasma was lower than in serum. However, since we only analyzed EDTA-based plasma, whereby EDTA itself could at least indirectly influence glucosidase activity, other types of plasma would still need to be examined comparatively. Whether 4-NPG or 4-MUG is used depends on factors such as sample type, hemolysis, and financial resources. The costs of 4-NPG are much lower than those of 4-MUG, although even when using 4-MUG, the total costs of the assay are relatively low.

Neutral α-glucosidase is most active in the pH range of 6.5–7.5. This has been demonstrated using serum and liver samples from pigs [17], but also for human serum [23]. Since activity is strongest at approximately pH 6.8, this pH value is often used in α-glucosidase assays [8,9,18,20,21,24]. However, pH 6.8 does not correspond to the physiological pH value in blood, which is why we always determined the α-glucosidase activity at pH 7.4 in the remaining tests. With this slightly higher pH value in the assay buffer, it is also less likely that any possible coactivity of the lysosomal (acid) α-glucosidase will be detected in blood. Other protocols add sodium dodecyl sulfate to the assay buffer to inhibit any possible lysosomal α-glucosidase activity [8,24,34]. We decided against it because sodium dodecyl sulfate often causes technical difficulties (i.e., foaming, precipitation). Studies by Alhadeff et al. have also shown that the pH ranges of lysosomal and neutral α-glucosidase in human serum are far apart [23].

Breeding- and season-dependent changes in the serum neutral α-glucosidase activity suggest a correlation between general metabolic activity of the body and α-glucosidase activity in blood fluid [20,21]. Nevertheless, it is unclear how quickly α-glucosidase activity changes in conjunction with general metabolic changes. Using serum samples taken from dairy cows around the calving period, and thus during their NEB period, we were able to show that neutral α-glucosidase activity is lowest when NEB is most pronounced. In this regard, blood glucose values were lowest five days after parturition but partially recovered some days later. The same changes have been observed for the serum α-glucosidase activity independently of the chromogenic substrate used for laboratory measurement. At the time of the lowest blood glucose levels *post partum*, other metabolic and hormonal parameters (i.e., BHB, NEFA, IGF-1, insulin) were significantly altered. Perinatal changes in these blood parameters are consistent with the findings of many other studies on this topic [27,35,36,37,38,39,40]. In addition, fructosamine levels in the blood also decrease *post partum*, but with a delay compared to blood glucose. This observation has also been made by others. [41,42]. Since fructosamine is the glycated form of albumin and other serum proteins, the delay is to be expected due to the half-life of serum proteins (21 days for albumin [43]).

The serum activity of neutral α-glucosidase changed peripartum in line with glucose levels rather than fructosamine levels. This suggests that α-glucosidase activity changes relatively quickly with the perinatal metabolic changes. In addition, α-glucosidase activity levels also reflected the adverse effect of parity and RFM complications on perinatal metabolism, with a high number of parities also being a significant risk factor for the occurrence of RFM [44]. The adverse effect of parity number or RFM on the degree of NEB after parturition is described well in other studies [30,35,45,46,47,48,49,50]. However, the perinatal blood profile of α-glucosidase activity in heifers still needs to be determined, as this was not investigated here.

What could be the cause of lower serum neutral α-glucosidase activity after parturition? Higher blood values of α-D-glucose, an inhibitor of neutral α-glucosidase activity [8], can be excluded as the blood glucose values are not increased but decreased *post partum*. We also used the enzyme substrates (4-NPG, 4-MUG) in excess compared to the blood glucose level, so that the activity values might primarily represent the blood α-glucosidase content. In this context, it is likely that less α-glucosidase is released into the blood from cells, particularly from secretory active cells. Neutral α-glucosidases are well expressed by nearly all cells and tissues [51]. As our measurement protocol used short synthetic substrates at neutral pH conditions, we mainly detected the activity of the type II neutral α-glucosidase of the endoplasmic reticulum [4,15]. Glucosidase II exists in two different isoforms (GANAB, GANC), which are expressed in a tissue-specific manner [51]. Regardless of the specific isoform, glucosidase II plays an important role in the process of protein N-glycosylation [4,15]. N-glycosylation is one of the most common post-translational protein modifications and occurs particularly in extracellular proteins.

Although it remains unclear which cell types mainly contribute to serum α-glucosidase levels, lower activity levels indicate either reduced formation and the release of N-linked glycoproteins or structural changes in the particular N-glycan residues. This is conceivable, as synthesis and post-translational modification of proteins always depend on chemical energy, which is less available *post partum* in dairy cows. In the blood, antibodies probably make up the majority of N-glycosylated proteins. As N-glycans contribute to the stability and function of antibodies [52], differences in their exact structures potentially could contribute, among other factors, to lower immune competence in the postnatal period of dairy cows [53]. Unfortunately, however, there are currently no studies that have investigated the glycosylation of antibodies in such an animal group.

What might be relevant reference values of α-glucosidase activity in bovine blood samples? The data of our study cannot give a clear answer to this question. In general, we observed an average 25% reduction in α-glucosidase activity in almost all dairy cows after calving compared to the prenatal phase, but each cow seems to have its own base value. Base value and/or average reduction may additionally be influenced by NEB prophylaxis (i.e., propylene glycol, Kexxtone^®^ bolus of high-risk cows). Moreover, the absolute α-glucosidase activity values depend on technical parameters (e.g., type of substrate, pH of the assay buffer, and method for calculating enzyme activity). Nevertheless, according to our protocol used in this and our previous study [21], we preliminarily suggest that serum neutral α-glucosidase levels below 3 U/L (4-NPG) or 10 U/L (4-MUG) indicate a calorie deficit in the animal. It is also noticeable that, in contrast to blood parameters such as NEFA, the intra-individual variations are smaller and therefore the data are normally distributed.

## 5. Conclusions

The blood α-glucosidase level has veterinary diagnostic potential and could be determined relatively easily and inexpensively by an enzymatic assay in a routine laboratory. In particular, α-glucosidase level could also be an alternative blood biomarker of NEB in dairy cows and most likely other livestock. Although the α-glucosidase activity does not necessarily better indicate NEB than known blood biomarkers, its NEB-related decrease provides new insights into metabolic changes during the NEB period. Moreover, our study raises new questions regarding the blood α-glucosidase values in dairy cows (e.g., effect of NEB prophylaxis, perinatal profile in heifers) and thus provides ideas for future studies.

## Figures and Tables

**Table 1:** Metabolism-related blood parameters in the dairy cow population studied.

Blood Parameter	14 d *Ante Partum*	5 d *Post Partum*
BHB (mmol/L)	0.92 ± 0.29	1.23 ± 0.72 ^a^
NEFA (mmol/L)	0.18 ± 0.13	0.61 ± 0.35 ^a^
IGF-1 (ng/mL)	259 ± 82.8	88.3 ± 79.0 ^a^
Insulin (µg/mL)	30.9 ± 15.2	9.21 ± 8.70 ^a^

Data are given as mean ± SD (n = 73 cows) with ^a^ *p* < 0.05 vs. 14 d *ante partum* (Wilcoxon signed-rank test).

**Table 2:** Characteristics of the dairy cow population depending on the number of parities.

Parity (n)	Animals (n)	Age (Months)	RFM (%)	Blood Parameters ^a^					
				BHB (mmol/L)	NEFA (mmol/L)	IGF-1 (ng/mL)	Insulin (µg/mL)	Glucose (mmol/L)	Fructosamine (mmol/L)
2	21	27 ± 6	10	1.08 ± 0.19	0.57 ± 0.17	125 ± 88.8	8.50 ± 14.8	3.29 ± 0.65	0.50 ± 0.19
3	22	42 ± 4	9	1.12 ± 0.39	0.40 ± 0.05	119 ± 45.8	10.8 ± 14.4	3.26 ± 0.95	0.50 ± 0.14
≥4 ^b^	30	65 ± 14 ^c^	40 ^c^	1.42 ± 0.19	0.79 ± 0.12 ^c^	42.0 ± 18.7 ^c^	8.49 ± 16.0	2.92 ± 1.12 ^c^	0.40 ± 0.14 ^c^

^a^ 5 d *post partum* except for fructosamine values (14 d *post partum*); ^b^ mainly 4th or 5th parity; ^c^ *p* < 0.05 vs. groups with less parities (chi^2^ test, ANOVA with Bonferroni test and, if the overall ANOVA procedure was not significant, multiple U tests between the parity groups, as appropriate).

## Data Availability

The raw data supporting the conclusions of this article will be made available by the authors on request.

## Abbreviations

The following abbreviations are used in this manuscript:

ANOVA	analysis of variance
AAG	lysosomal α-glucosidase, acid (gene GAA)
BCS	body condition score
BHB	β-hydroxybutyric acid
ER	endoplasmic reticulum
MGAM	maltase-glucoamylase, intestinal
4-MUG	4-methylumbelliferyl α-D-glucopyranoside
4-MU	4-methylumbelliferon
NAG-AB	tissue α-glucosidase, neutral AB (gene GANAB)
NAG-C	tissue α-glucosidase, neutral C (gene GANC)
NEB	negative energy balance
NEFA	non-esterified fatty acids
4-NPG	4-nitrophenyl α-D-glucopyranoside
4-NP	4-nitrophenol
OD	optical density
RFM	retained fetal membrane
U	Unit (enzymatic activity)
